# Negative regulation by nuclear receptors: a plethora of mechanisms

**DOI:** 10.1016/j.tem.2010.11.004

**Published:** 2011-03

**Authors:** Guilherme M. Santos, Louise Fairall, John W.R. Schwabe

**Affiliations:** 1 MRC Laboratory of Molecular Biology, Hills Road, Cambridge, CB2 0QH, UK; 2 Henry Wellcome Laboratories of Structural Biology, Department of Biochemistry, University of Leicester, Lancaster Road, Leicester, LE1 9HN, UK

## Abstract

Nuclear receptors are arguably the best understood transcriptional regulators. We know a great deal about the mechanisms through which they activate transcription in response to ligand binding and about the mechanisms through which they repress transcription in the absence of ligand. However, endocrine regulation often requires that ligand-bound receptors repress transcription of a subset of genes. An understanding of the mechanism for ligand-induced repression and how this differs from activation has proven elusive. A number of recent studies have directly or indirectly addressed this problem. Yet it seems the more evidence that accumulates, the more complex the mystery becomes.

## Classical repression by unliganded nuclear receptors

**Figure 1:**
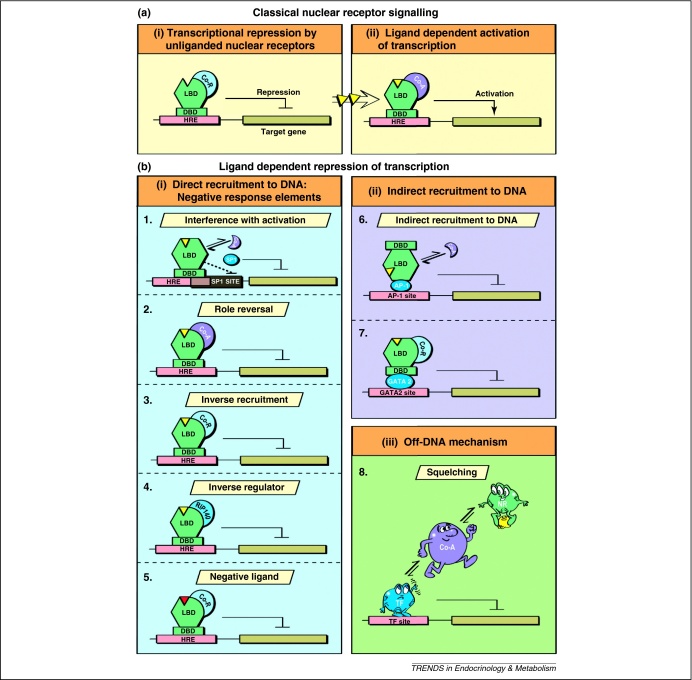
Diverse modes of signalling by nuclear receptors. (**a**) Classical nuclear receptor (NR) signalling. **(i)** Transcriptional repression through recruitment of corepressor complexes to unliganded nuclear receptors. **(ii)** Transcriptional activation through recruitment of coactivators to ligand-bound nuclear receptors. (**b**) Ligand-dependent repression of transcription. **(i)** Negative response element: direct recruitment to DNA. (1) Nuclear receptor interference with the activation of transcription by other factors. For example, ligand-bound TR prevents the general transcriptional activator SP1 from binding to the β-amyloid precursor gene. (2) Coactivator role reversal leading to transcriptional repression. For example, SRC1 contributes to repression by ligand-bound TR. (3) Inverse recruitment of corepressors to ligand-bound receptors. For example, the corepressor NCoR has been implicated in association with ligand-bound TR on the gene encoding TSHα. (4) Factors such as RIP140 act as inverse regulators because they serve as corepressors yet are recruited to ligand-bound receptors. (5) Synthetic and natural inverse agonists serve as negative ligands because they promote recruitment with corepressor complexes. For example, haem-binding promotes repression by REV-ERB. **(ii)***Trans*-repression by ligand-bound receptors. (6) Ligand-bound GR interacts with and prevents activation of AP1-mediated transcription. (7) Ligand-bound TR contributes to repression of the gene encoding TSHβ through interaction with the GATA2 transcription factor. **(iii)** Downregulation through off-DNA mechanisms. (8) Genome-wide studies suggest that many downregulated genes do not directly recruit nuclear receptors. Hence, the downregulation observed is likely to be due to squelching effects.

The first insights into how nuclear receptors repress transcription in the absence of their activating ligands were obtained through cloning of the corepressor proteins silencing mediator of retinoid and thyroid hormone receptor (SMRT) [1] and nuclear receptor corepressor (NCoR) [2] that interact with many unliganded nuclear receptors. It is now known that corepressor proteins exist as large multiprotein complexes containing enzymes such as histone deacetylases that repress transcription by regulating the nature of the chromatin local to the gene promoter. Corepressors are usually released on ligand binding through changes in the conformation and dynamics of the receptor ligand-binding domain, which favour recruitment of coactivator proteins (Figure 1) [3]. An important feature of these interactions is that corepressors and coactivators bind to overlapping surfaces on the nuclear receptors such that their binding is mutually exclusive. This is important because competition between coregulator proteins can play a key role in the tissue-specific regulation of some target genes [4].

Although the classic activity of most ligand-bound nuclear receptors activates transcription, ligand-bound nuclear receptors repress the transcription of certain genes, a process termed negative regulation. The classic example of this is repression of thyroid-stimulating hormone (TSHα) by the thyroid receptor (TR) in the presence of T3. It is now known that such negative regulation occurs with many other nuclear receptors and genes.

Over the years, various mechanisms have been suggested to explain how ligand-dependent repression occurs (Figure 1). Here we discuss these different mechanisms and examine how they can be reconciled with recent genome-wide studies.

## Negative response elements

A relatively long-standing idea in the nuclear receptor field is the concept of negative response elements. This concept emerged when it became clear that ligand-bound glucocorticoid (GR) and TR receptors downregulate specific target genes, which raised the question as to what mechanism might explain how ligands could have opposite transcriptional effects on certain genes. Studies of GR downregulation of the prolactin gene revealed that ligand-bound GR, associated with a negative glucocorticoid response element (nGRE), acts through the ‘reversal of a constitutive enhancer activity’ [5]. The authors speculated that when ligand-bound GR binds to nGREs, its conformation does ‘not support’ transcriptional activation. However, the molecular mechanism underlying this phenomenon remained uncertain.

Subsequent studies investigating downregulation of the genes encoding TSHα [6] and TSHβ [7] identified a negative thyroid hormone response element (nTRE) in the proximal promoter, between the TATA box and the transcriptional start site of the gene. It was proposed that downregulation was the result of steric interference with other components of the transcriptional machinery.

Intriguingly, examination of the DNA sequence of both nGRE and nTRE suggested that the sequences are significantly divergent from the classical positive response elements that mediate transcriptional activation by these receptors, which brought into doubt whether or not receptors actually bind to negative response elements. This issue was resolved using a TRβ mutant defective in DNA binding, which was unable to suppress expression of *TSH* genes in response to T3 in both cell-based assays [8] and knock-in mice [9]. Thus, these findings demonstrated that DNA-binding activity is necessary to downregulate *TSH* genes.

Support for the idea that negative response elements function through promoter interference mechanisms came from further studies of the prolactin and β-amyloid precursor genes. It was shown that a GR binding site (nGRE) in the prolactin promoter overlaps with the binding sites for other transcription factors, including Oct-1 and Pbx. Addition of an isolated GR DNA-binding domain to the nuclear extract precluded DNA binding of both Oct-1 and Pbx [10], which implies that direct competition exists for DNA-binding on this promoter. Analogously, the binding sites for TR and the transcription factor SP1 overlap each other in the β-amyloid precursor protein (APP) promoter [11]. T3 binding to TR increases the DNA-binding affinity of TR, preventing SP1–DNA complex formation and consequently downregulating SP1-dependent expression of APP. Together, these findings suggest an interplay between competing transcriptional regulators on certain promoters. Consequently, ligand-bound nuclear receptors can block transcriptional activation by other factors, which leads to downregulation of these genes.

## Role reversal of coregulators

**Figure 2:**
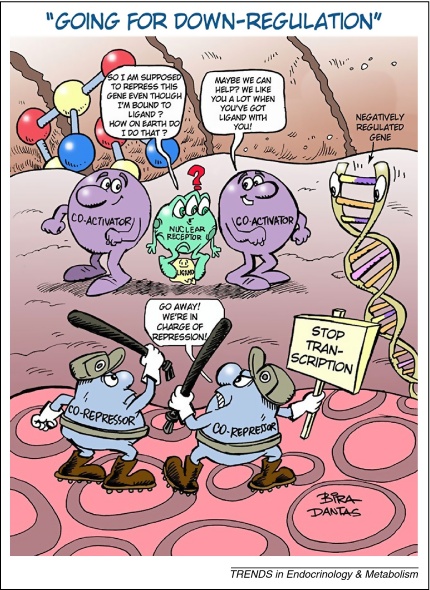
The conundrum of downregulation by ligand-bound nuclear receptors. When ligand-bound nuclear receptors negatively regulate target genes, many of the classical principles of nuclear receptor signalling are reversed.

Since the early studies of negative response elements, we have learnt that nuclear receptors (like most transcription factors) regulate gene expression through the recruitment of large complexes containing a variety of coregulators and effector enzymes that target chromatin and other factors. As discussed, in the classical activity of nuclear receptors, ligand-bound receptors recruit coactivator complexes and unliganded receptors bind corepressor complexes. This of course raises the question as to what type of coregulator complex is recruited by liganded receptors on a negatively regulated promoter. This is a difficult problem because multiple studies have explained how ligand binding to nuclear receptors promotes interaction with coactivator complexes containing histone acetylases (and methyl transferases) and displaces corepressor complexes associated with histone deacetylases (and demethylases). So what explains downregulation by ligand-bound receptors? Do they somehow recruit corepressors on negatively regulated genes? Or do histone acetylases somehow repress these genes? This conundrum is illustrated by the cartoon in Figure 2.

An early study of negative regulation of the gene encoding TSHα provided evidence that the role of coregulators might be reversed on negatively regulated genes, because recruitment of corepressors to the gene encoding TSHα was associated with activation [12]. The reversal of action seems to lie in the finding that corepressor recruitment to this gene results in histone acetylation. Similarly, the corepressor SMRT mediates an increase in transcription at the nTRE within the Rous sarcoma virus long terminal repeat of TSHα [13]. In this case, protease digestion and mobility shift assays suggested that the TR–SMRT complexes had different conformations depending on whether they were bound to a negative or positive hormone response element. Further studies lend support to the concept of coregulator role reversal depending on the particular response element. TR mutants defective in corepressor recruitment no longer activate an nTRE present in the *SOD1* promoter. Conversely, a receptor defective in coactivator recruitment, but still able to interact with corepressors, shows impaired downregulation in response to thyroid hormone [14,15].

The role of coactivators in mediating repression is supported by several studies. Mice lacking the steroid receptor coactivator-1 (SRC1) revealed a role for this coactivator in activating some liganded TR-responsive genes and also repressing transcription from liganded and unliganded TR-responsive genes [16,17]. Similarly, the coactivator SRC3 functions as a repressor in lymphocytes [18].

Taken together, these findings suggest that both promoter and cellular context can determine whether a particular coregulator acts as an activator or a repressor. Two aspects of these role reversals remain unclear. First, what is it about a particular promoter element or cellular environment that results in coregulator role reversal? Second, how are such role reversals implemented by the coregulator complexes?

Evidence that the sequence of DNA response elements can influence transcriptional outcome came from studies of various GR binding sites that seem to require or exploit different activation domains within the receptor [19]. Indeed, a single base-pair change in the DNA of the response element influences GR conformation [20]. Thus, it seems that DNA serves as a sequence-specific allosteric ligand that modulates the regulatory activity of the GR.

The histone demethylase LSD1 illustrates a well-established example of the mechanism through which the role of a coregulator can be reversed. LSD1 normally acts as a corepressor when recruited to chromatin as part of the CoREST complex. However, LSD1 can act as a coactivator of the androgen receptor [21]. The mechanism for this switch in activity has recently been established. When acting as a corepressor, LSD1 demethylates lysine 4 on histone 3 (H3K4). When histone 3 is phosphorylated (H3T6) by PKCβ kinase, which is recruited by the androgen receptor, then LSD1 demethylates H3K9 and leaves methyl groups on H3K4, which leads to activation of transcription [22]. This illustrates how a simple post-translational modification can lead to reversal of transcriptional activity.

It seems likely that covalent modifications, as well as differential splicing of coregulators, will explain many examples of coregulator role reversal. In addition to a large repertoire of splice variants, coregulator proteins are extensively modified by acetylation, phosphorylation, ubiquitination, methylation and SUMOylation [23–26]. Together, these variations give enormous scope for fine-tuning of the transcriptional outcome.

## Inverse recruitment of corepressors

Role reversal of coregulators (e.g. a corepressor acting as a coactivator) is now well established. However, it was recently shown that another mechanism plays a role in negative regulation. In this case, it seems that a ligand-bound TR can repress genes by recruiting the corepressor NCoR. This can be thought of as inverse recruitment of coregulators. The evidence for this arises from a recent study in mice containing a mutant corepressor, NCoR, harbouring mutations in the deacetylase activation domain (DAD), which abrogate interaction with the deacetylase HDAC3. In these mice, several TH-responsive genes are modestly activated in the absence of TH, which suggests that failure to recruit HDAC3 leads to a failure of normal gene repression. However, more surprisingly, several genes that are normally repressed by ligand-bound TH are activated in the mutant mice [27]. This suggests that on positively regulated genes, NCoR is displaced on ligand binding to the TR, which allows recruitment of coactivators, but on the negatively regulated TSHα promoter, NCoR is recruited to the ligand-bound TR, which leads to transcriptional repression. It remains to be understood through what mechanism this inverse recruitment of corepressors on negatively regulated genes might be achieved.

## Inverse coregulators recruited by normally activating ligands

The concept of coregulator role reversal derives from the finding that coregulators that normally bring about one outcome, can in certain circumstances, bring about the opposite outcome. At least one coregulator has been identified that seems to be the extreme example of role reversal. Receptor interacting protein of 140 kDa (RIP140) is a coregulator with multiple interaction motifs that allow it to be recruited to ligand-bound receptor [28]. However, RIP140 acts as a corepressor protein that recruits histone deacetylase enzymes through several complexes [29]. Thus, RIP140 can be considered an inverse coregulator. Its biological role seems to be regulation of metabolism by balancing the activities of the conventional coactivator PGC1α [30]. Similarly, LCoR is a corepressor that is recruited to ligand-bound oestrogen receptor α (ERα). Like RIP140, LCoR recruits other repressor molecules such as the C-terminal binding protein and histone deacetylases [31].

## Inverse agonists promote corepressor recruitment

Nuclear receptors are important targets for pharmaceutical intervention and there has been much effort to develop agonists to activate the receptor and antagonists to compete with and block the activation activity of the natural ligand. Importantly, a third type of synthetic ligand for nuclear receptors has emerged, inverse agonists. These pharmaceutical ligands bind to nuclear receptors and promote the recruitment of corepressor complexes, which leads to active repression of target gene transcription in response to ligands. One example of an effective pharmacological inverse agonist is tamoxifen, which binds to the oestrogen receptor, promotes recruitment of corepressors such as NCoR, and represses oestrogen receptor target genes.

It has only been relatively recently recognized that naturally occurring ligands can act as inverse agonists. REV-ERBα and β were originally described as orphan receptors that seemed to function as constitutive transcriptional repressors, directly binding to DNA and recruiting the NCoR corepressor [32]. The lack of activation was explained by sequence analyses that suggested that they lack the carboxy-terminal helix of the ligand-binding domain, which is essential for coactivator recruitment by other nuclear receptors. Recent studies have revealed that haem serves as a regulatory ligand for REV-ERB. However, haem binding does not activate the receptor, but instead increases the affinity for the NCoR corepressor, which in turn enhances the repression activity [33].

Like REV-ERB, no regulatory ligand for RORβ had been identified. However, the crystal structure of RORβ revealed a fatty acid ligand (stearate) in the ligand-binding pocket. Surprisingly, the steric acid ligand does not seem to activate or antagonize RORβ transcriptional activity [34]. More recently, however, it was found that RORβ binds all-*trans* retinoid acid, which downregulates the transcriptional activity of RORβ [35]. Thus, it seems that retinoic acid might act as an inverse agonist of RORβ.

Another receptor that seems to be regulated by a natural inverse agonist is the constitutive androstane receptor (CAR), a xenobiotic-responsive transcription factor. This receptor, as suggested by its name, has strong transcriptional activity in the absence of ligand. This constitutive activity is explained by some structural differences that lock the receptor in an active conformation [36]. Some years ago, it was shown that the androstane metabolite (androstanol) acts as an inverse agonist and reverses the constitutive activity of CAR [37]. The crystal structure of CAR with androstenol shows that this inverse agonist binds within the ligand-binding pocket and locks the receptor in an inactive conformation that does not support coactivator binding, but instead allows the recruitment of corepressors [38,39].

## Indirect repression by ligand-bound nuclear receptors

Nuclear receptors often engage in crosstalk with other transcriptional regulators, and in many cases this results in repression of the activity of other factors. This is termed *trans*-repression and is commonly dependent on ligand binding to the nuclear receptor. One of the earliest examples of this involved an interaction between the ER and the tissue-specific transcription factor Pit-1, which leads to downregulation of the prolactin gene [40].

Later, using different *in vivo* strategies, a model was proposed for such *trans*-repression by GR that involves tethering of the receptor by direct binding to other transcription factors such as NF-κB [41] and AP1 [42,43]. More recently, it was demonstrated for AP1 transcription factors that only dimers containing FOS are *trans*-repressed by GR. Thus, the dimer composition of AP1 can regulate the positive and negative transcriptional activity [44]. In this type of *trans*-repression mechanism, it seems that the nuclear receptor itself does not bind directly to DNA and indeed in some cases the DBD is not needed for *trans*-repression to occur [40,42]. However, in other cases the nuclear receptor DBD does seem to be required, which suggests that the DBD plays a role in the interaction with other transcription factors [45–47].

Recent experiments on negative regulation of the gene encoding TSHβ suggest that crosstalk occurs between ligand-bound TR and the transcription factor GATA2 [48]. The Zn-finger region of GATA2 interacts with the TR DBD, and this complex is required for negative regulation by thyroid hormone. In this case, the effect of the ligand perhaps controls the differential affinity of TR for the GATA2-RE and the negative regulatory element [49].

Another important example of *trans*-repression is mediated by PPARγ. Ligand-dependent SUMOylation of the PPARγ ligand-binding domain results in PPARγ recruitment of the corepressor (NCoR)–histone deacetylase-3 (HDAC3) complex to inflammatory gene promoters [50].

## Insights from genome-wide studies

Genome-wide studies of transcription have dramatically changed our view of transcriptional repression by nuclear receptors. Although we used to believe that downregulation of genes in response to nuclear receptor ligands was a relatively minor affair, it turns out that as many as half the genes regulated by nuclear receptor ligands are in fact downregulated [51]. Clearly in some cases this will be a secondary effect of upregulation of another factor such as a repressive transcription factor or even a corepressor protein. However, it seems that much of the downregulation by nuclear receptor ligands occurs on the same time scale as upregulation, which argues against secondary effects being responsible for this downregulation. How can this large-scale downregulation be explained?

Microarray-based gene-expression profiling experiments have identified genes that are either up- or downregulated by nuclear receptors. Analysis of these experiments together with ChIP-on-chip and ChIP-Seq experiments revealed the location of the binding sites for nuclear receptors. What has emerged from these studies is that the majority of upregulated genes, both for the ER [52] and GR [53], are associated with binding sites for the receptor. In stark contrast, few of the downregulated genes seem to be located in realistic proximity to binding sites for the receptors. Thus, it seems that much of the downregulation observed occurs without interaction between the nuclear receptor and a promoter or enhancer sequence close to the regulated gene.

To explain this, we must give some thought to the inherent transcriptional potential of the cell at any one time. If we assume that there is a maximum amount of transcription a cell can perform, perhaps through limited concentrations of certain components of the transcriptional machinery, then a sudden upregulation of one set of genes in response to an activating ligand will necessarily result in downregulation of other genes. This phenomenon is well known in the transcription field as squelching and is commonly observed when a transcriptional regulator is overexpressed [54]. The excess non-DNA-bound material titrates out other components of the transcriptional machinery and hence causes downregulation of other genes because the squelched components become limiting. If squelching is really the explanation for this large-scale downregulation of gene expression in response to activating nuclear receptor ligands, we must ask whether this downregulation is physiologically important.

A recent puzzle has emerged through mapping of the genome-wide locations of histone acetyltransferases (HATs) and HDACs. These genome-wide studies revealed that not only are HATs associated with actively transcribed genes, but HDACs (well-known components of repression complexes) are also found almost exclusively at active genes [55]. This suggests that we need to completely reconsider the roles of the coregulators. One explanation, of course, is that active genes are associated with acetylated histones. Acetylated histones are substrates for HDACs, so perhaps it is natural that HDACs should be at active genes. Indeed, a study examining ERα-induced expression of the gene encoding PS2 revealed that transcriptional activation is a cyclical process in which recruitment of ERα and proteins involved in activation and repression cycle approximately every 40 min [56]. Acetylation, deacetylation, methylation and demethylation of histones H3 and H4 also followed a cyclical pattern. These findings suggest that corepressor recruitment might be an essential part of transcriptional activation by serving to reset the transcriptional machinery.

## Perspectives

Over the last 25 years of studies of transcription regulation by nuclear receptors, many overarching general principles have emerged that have stood the test of time. These include the concepts of: (i) DNA response element recruitment of receptors to promoters or enhancers of target genes; (ii) binding of ligands to nuclear receptors to control the recruitment of coregulator complexes; and (iii) implementation by coregulator complexes containing histone-modifying enzymes of a histone code that directs transcriptional activity of target genes by modifying the structure of chromatin. However, as the details have been explored, it has emerged that each of these broad concepts encompasses an enormous range of diversity with multifunctional complexes and outcomes. At one time, downregulation of genes in response to nuclear receptor ligands seemed to conflict directly with the established principles. Now, set in the context of the widely diverse details of gene regulation, this downregulation no longer seems so surprising, nor should it be unexpected that many different mechanisms contribute to this ligand-dependent downregulation.

## Glossary

Downregulation: reduction in the rate of transcription to a level below that which would otherwise be observed. The opposite is termed upregulation.
Impairment of activation: prevention of or reduction in the activation of transcription by other factors.
Inverse regulation: activation of transcription by nuclear receptors in the absence of ligand, and downregulation in the presence of ligand.
Negatively regulated genes: genes that are downregulated under conditions that would normally be expected to increase their rate of transcription.
Repression: reduction in the rate of transcription to a level lower than basal through the recruitment of repressive factors or complexes.
Squelching: indirect interference in the transcription of a gene through the sequestering of factors normally required for its transcription. This can result in down- or upregulation of the gene in question.
